# Contradiction and Complacency Shape Attitudes towards the Toll of Roads on Wildlife

**DOI:** 10.3390/ani6060040

**Published:** 2016-06-17

**Authors:** Daniel Ramp, Vanessa K. Wilson, David B. Croft

**Affiliations:** 1 School of Biological, Earth & Environmental Sciences, University of New South Wales, Sydney, NSW 2052, Australia; sandvwillow@gmail.com (V.K.W.); d.croft@unsw.edu.au (D.B.C.); 2 Centre for Compassionate Conservation, School of Life Sciences, University of Technology Sydney, Broadway, NSW 2007, Australia

**Keywords:** road ecology, driver behaviour, driver attitudes, wildlife vehicle collisions, road-kill

## Abstract

**Simple Summary:**

Mitigating the toll of roads on wildlife can become difficult when awareness and exposure does not result in willingness to change driving behaviour. Using a self-reporting questionnaire, we found that while most drivers view wildlife vehicle collisions as a serious issue, increasing exposure to collisions decreased this attitude and it did not translate into willingness to adopt additional mitigation strategies. In addition, despite most respondents stating they routinely drive slower when collision risk is high (at dusk and dawn), our assessment of driving trends via traffic speeds suggested this sentiment was not generally adhered to. We suggest that competing priorities and complacency when risk to people is low may adversely affect willingness to prevent collisions.

**Abstract:**

Most people in the world now live in cities. Urbanisation simultaneously isolates people from nature and contributes to biodiversity decline. As cities expand, suburban development and the road infrastructure to support them widens their impact on wildlife. Even so, urban communities, especially those on the peri-urban fringe, endeavour to support biodiversity through wildlife friendly gardens, green spaces and corridors, and conservation estates. On one hand, many who live on city fringes do so because they enjoy proximity to nature, however, the ever increasing intrusion of roads leads to conflict with wildlife. Trauma (usually fatal) to wildlife and (usually emotional and financial) to people ensues. Exposure to this trauma, therefore, should inform attitudes towards wildlife vehicle collisions (WVC) and be linked to willingness to reduce risk of further WVC. While there is good anecdotal evidence for this response, competing priorities and better understanding of the likelihood of human injury or fatalities, as opposed to wildlife fatalities, may confound this trend. In this paper we sought to explore this relationship with a quantitative study of driver behaviour and attitudes to WVC from a cohort of residents and visitors who drive through a peri-urban reserve (Royal National Park) on the outskirts of Sydney, Australia. We distributed a self-reporting questionnaire and received responses from 105 local residents and 51 visitors to small townships accessed by roads through the national park. We sought the respondents’ exposure to WVC, their evasive actions in an impending WVC, their attitudes to wildlife fatalities, their strategies to reduce the risk of WVC, and their willingness to adopt new ameliorative measures. The results were partitioned by driver demographics and residency. Residents were generally well informed about mitigation strategies but exposure led to a decrease in viewing WVC as very serious. In addition, despite most respondents stating they routinely drive slower when collision risk is high (at dusk and dawn), our assessment of driving trends via traffic speeds suggested this sentiment was not generally adhered to. Thus we unveil some of the complexities in tackling driver’s willingness to act on reducing risk of WVC, particularly when risk of human trauma is low.

## 1. Introduction

Urbanisation and the dissection of landscapes by roads increases apace as the human population grows and rural populations shift to cities. Although some wildlife adapt and can flourish in urban landscapes [1], biodiversity loss is often stark. City dwellers can experience a disconnect from nature [2], prompting some to either seek out wild experiences by visiting national parks and reserves or by choosing to live in areas that provide a measure of urbanity and connectedness to fringe (peri-urban) habitat. Activities to encourage wildlife into urban and peri-urban landscapes are popular, including the establishment of wildlife friendly gardens, green spaces and biodiversity corridors. However, as cities expand, suburban development and the road infrastructure to support them increases the toll on wildlife. The ever growing intrusion of roads leads to conflict with wildlife and creates substantial trauma to animals (usually fatal) and people (usually emotional and financial, but sometimes fatal). Interactions between people and other animals are often conflicted by a variety of competing priorities [3], yet animal needs are almost always subjugated by our own, whether consciously or not [4].

The effect of roads on wildlife has evolved into the discipline of ‘road ecology’ [5]. There have been significant advances in identifying collision hotspots, the causes of wildlife vehicle collisions (WVC), and the ways in which these can be ameliorated, from the perspectives of both wildlife biologists and road engineers [6,7]. Animal movement patterns, density and road conditions are key determinants of WVC [8], however, driver behaviour, exposure and attitudes are also important and can strongly influence collision likelihood [9]. Calls for systems to increase the awareness of drivers are common [10,11] but most research has examined the response of drivers to animal-collision warning signs [12,13] and animal detection systems [14]. Wildlife warning signs are often used as a low cost solution but significant reductions in WVC have rarely been demonstrated [15]. Nonetheless, it is known that drivers can effectively reduce the likelihood of WVC by reducing their driving speed and remaining alert while driving through areas where animals are more abundant [11,16]. One study reported that about 60% of WVC result from inappropriate driver reaction [17]. Drivers need to make complex decisions taking into account the likely and actual behaviour of the animal (e.g., see studies on kangaroos [18]) in an impending collision and the actions and opportunities to evade impact.

Aside from wildlife-friendly road design, the key to reducing WVC is making drivers aware of the animals likely to intrude onto roads and of appropriate responses when animals are encountered [19]. Around 90%–95% of traffic accidents are the result of human actions, either solely or in conjunction with other factors [20]. Research has examined the link between behaviour and traffic crashes primarily through self-reporting driver behaviour questionnaires [21,22,23] as they provide a satisfactory assessment method where systematic data are unavailable [24]. This research has focussed on identifying aberrant behaviour that leads to traffic accidents, such as violations of road laws, driving mistakes, inattention and lack of suitable driving experience [23]. Thus it seems fruitful to utilise a similar framework to investigate driver behaviour and attitudes towards WVC. However, although exposure, defined here as direct experience of WVC and resultant vehicle damage and injury [25], and attitudes to potentially traumatic encounters with animals on roads is important, willingness to make appropriate behavioural adjustments to minimise such trauma is likely to be affected by multiple costs and benefits. For example, it is possible that awareness of collision risk does not necessarily result in appropriate behavioural adjustments because familiarity and habituation can breed complacency when competing costs are considered or risks are low. This situation may arise when outcomes of collisions only rarely result in vehicle damage or injury to occupants (e.g., when body weights of the animals involved are below 25 kg) or when roads are travelled frequently.

The aim of this study, therefore, was (a) to assess how exposure, experience and attitude affects decision making at collision moments; (b) to determine whether increased exposure and driving experience positively shapes the attitudes of drivers towards WVC; (c) to assess how exposure, experience and attitude affects the willingness to adopt additional collision mitigation measures; and (d) to assess whether driver actions matched attitudes and exposure. The focus was a conservation reserve, Royal National Park (RNP), on the outskirts of Sydney, Australia, that contains two townships (and thus local residents), is a popular tourist destination for local and international visitors, and incurs a large number of animal-vehicle collisions with primarily small to medium-sized animals [26]. Our approach was to combine a self-reporting driver behaviour questionnaire, distributed to both residents and visitors to the Park, with speed monitoring conducted at two locations designed to distinguish between resident and visitor driving behaviour. Hence, this study location enabled partitioning of respondents into groups with contrasting familiarity of the survey roads and associated collision risk.

## 2. Materials and Methods

### 2.1. Study Location

Royal National Park (34°05′S, 151°05′E) is located on Sydney’s southern outskirts in New South Wales, Australia. It is the second oldest national park in the world (established in 1879) and forms a 15,068-ha peri-urban reserve on the southern outskirts of Australia’s largest city (Sydney, population 4.2 million). In this location, it is encroached upon by urban development to the north (although it is protected somewhat by Port Hacking) and fragmented by the Princess Highway along the western border. Even so, it has a wide diversity of habitats including heathland, woodland, eucalypt forest, rainforest, wetland and swamps, which support more than 50 mammal species [27]. The bird life is diverse with 241 species, and includes a number of vulnerable and endangered species. There are about 40 species of reptile and 30 species of amphibian. The climate is temperate, where average day-time temperatures range from around 7 to 17 °C in the coolest month of July, to 18 to 26 °C in the warmest month of January. Average rainfall is highest (126 mm) in June and lowest (62 mm) in September (recorded at Sydney Airport by the Australian Bureau of Meteorology).

The park contains two townships (Bundeena and Maianbar) within its borders that provide residence to over 2000 people and also has over one million visitors annually. It has a network of paved roads with posted speed limits of mostly 60 and 80 km·h^−1^ The major road out of the park, Farnell Avenue, typically has an annual average of 3000 vehicles per day [26]. The road network has a significant impact on the fauna as 58% of the park is within 1 km of a paved road, while 86% of the park is within 500 m of any type of road (including un-surfaced roads and fire trails). Of 112 animal carcases identified in a survey on or near roads over 143 days, conducted from the entrance of the Park to the township of Bundeena, most were of small birds (66%) or small to medium sized mammals (32%, primarily swamp wallabies *Wallabia bicolor*), with only 3% representing animals likely to cause significant vehicle damage or injury (Rusa deer *Cervus timorensis*) [26].

### 2.2. Driver Questionnaire

We asked 14 questions of drivers using the park in order to assess their attitudes to WVC in relation to their previous experiences with animals on roads, and their demography (see Appendix A for questionnaire). The first four questions related to the demography and experience of drivers. Drivers were identified as either residents living within one of the two townships or as visitors to the park. The next eight questions examined the driver’s experience with WVC (colloquially referred to as road-kill), the type of fauna hit, the driver’s response to an impending collision, the driver’s response to the welfare of the animal if a collision occurred, the counter-measures employed on the vehicle, and the damage sustained by the vehicle. The remaining two questions were used to ascertain the general attitudes of the drivers to WVC and what mitigation strategies they would adopt (if any).

### 2.3. Subjects

Questionnaires were distributed and collected from 13 locations in Bundeena (10 local service businesses) and the Royal National Park (entrance booth, visitors centre, café) with the assistance of personnel from the businesses and the NSW Office of Environment and Heritage. Respondents were offered an incentive to complete the survey instrument through a voluntary entry into a competition for one of five (donated) prizes (valued from $15 to $80). The questionnaires were circulated from the 8 August to the 30 September 2003 when the competition was drawn. Respondents were instructed to complete the questionnaire in their own time and deposit it by 30 September in a collection box at one of the 13 distribution locations.

### 2.4. Driver Demography

A total of 156 surveys were completed and returned: 105 from residents and 51 from visitors. The responses are summarised across the categories of driver residency, age (pooled into young, middle-aged, old), and experience with animal collisions (Appendix B). The age categories of resident and visitor respondents were similar (*χ*^2^ = 3.48, *df* = 6, *p* = 0.74), with approximately 60% between the ages of 30 and 49 years of age. Likewise, the vehicle type driven by residents and visitors was similar, with most (~50%) respondents driving sedans and a further 25% driving 4WDs.

### 2.5. Driver Speed

**Figure 1:**
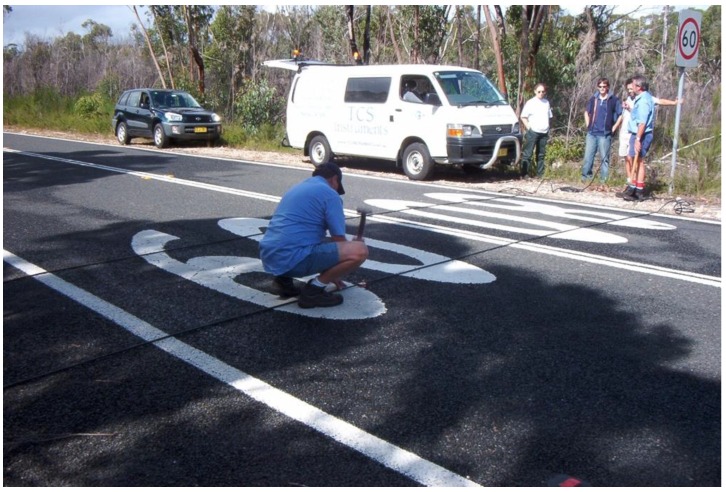
Vehicle speed technology was deployed on Farnell Avenue (seen below, photo Daniel Ramp) near the entrance to the Royal National Park, Australia, and on Bundeena Drive near the township of Bundeena. Twin tubes on the road surface connected to data loggers were deployed for a period of 143 days and recorded vehicle speeds and type in both directions.

To determine what drivers do relative to what they say they do or would do in relation to mitigating WVC, we measured vehicle speeds on Bundeena Drive and Farnell Avenue in both directions as a function of the posted limit, the traffic volume and the time of day (Figure 1). The Bundeena Drive location was used to infer travel in (north) and out (south) of Bundeena township, while the Farnell Avenue location was used to capture all drivers entering (east) or leaving (west) the national park. The methods and analysis are discussed in detail in [26]. Here we present a summary that includes the mean hourly vehicle speed (km·h^−1^) averaged over the period of study (10 April and 31 August 2003) and the average maximum hourly speed (km·h^−1^) for the study period to relate driver attitudes to an important aspect of driver behaviour.

### 2.6. Statistical Analysis

The responses to the questionnaires were summarised using SPSS for Windows v13.0 (SPSS Inc., Chicago, IL, USA), where all chi-squared tests reported used exact estimation methods. Testing of key questions were conducted in R v3.2.4 [28]. Driving experience was simply defined as the number of years spent driving. There are different mechanisms for determining exposure to the risk posed by wildlife on roads [25], however, for simplicity we used a measure of accident experience. Exposure was calculated by taking the mean response of the number of collisions and the seriousness of the damage experienced. This was achieved by converting responses into integers 0, 1, 2 or 3, where 0 reflected no collisions or damage and 3 reflected greater than 10 collisions and where vehicles were written-off. Attitude was defined from question 13 and was similarly converted into integers 0, 1, 2 or 3, where 0 reflected no problem and 3 reflected seeing road-kill as a very serious issue. No respondents selected the “indifferent” category and was removed from analysis as it was not sufficiently different from “it’s no problem at all”. Binary logistic regressions were constructed to examine (a) current mitigation strategies of drivers, defined as dummy variables because actions were not mutually exclusive (braked, accelerated, dimmed lights, honked horn or took no action), at the collision moment. Ordinal logistic regressions (run using the “*polr*” function in the MASS library in R) were conducted to assess (b) the influence of exposure, experience and driver residential status on driver attitudes towards road-kill; and (c) the influence of exposure, experience, attitude and driver residential status on the willingness to adopt additional collision mitigation strategies. Graphical representation of the relationships were produced by creating simulation datasets where all bar the variable of interest were held at their mean values.

## 3. Results

### 3.1. Driver Experience with WVC

**Figure 2:**
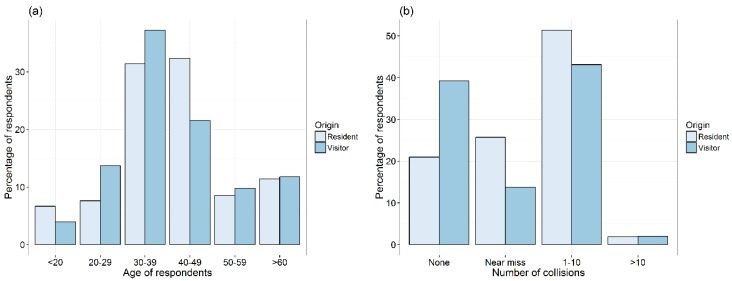

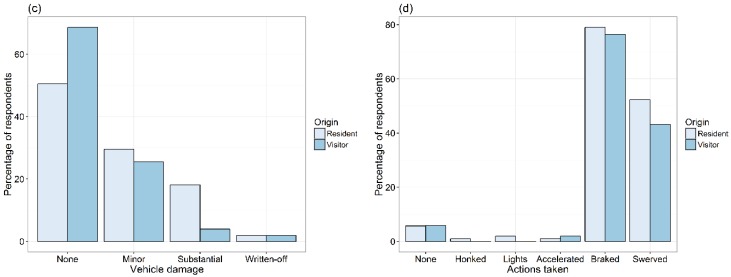
The percentage of resident and visitor respondents (dark grey and grey respectively) to the driver behaviour questionnaire in the Royal National Park, Sydney, Australia for (**a**) each of six age categories; (**b**) each of four categories of the number of reported collisions; (**c**) each of four categories of the most vehicle damage experienced; and (**d**) each of six categories of the most common actions taken by drivers in response to observed animals on roads.

The most common road-kill experience was to have hit between 1 and 10 animals (49% total) with a significantly higher proportion of residents experiencing near misses or 1–10 animals hit than visitors (*χ*^2^ = 9.06, *df* = 4, *p* = 0.045) (Figure 2b). Many respondents (44% total) claimed to have sustained damage to their vehicles in a collision or near collision with an animal. Residents claimed more damage to their vehicles than visitors (49.5% *vs.* 31.4%) but the frequencies of damage types were not significantly different between residents and visitors (*χ*^2^ = 6.87, *df* = 4, *p* = 0.12) (Figure 2c). Four residents and one visitor reported human injuries as a result of their WVC experiences.

### 3.2. Driver Behaviour to Mitigate WVC

The most common avoidance reactions were braking (78%) followed by swerving (49%) (Figure 2d). The probability that the driver braked or swerved was not a significant function of residency, driver age, vehicle type, driving experience, previous encounters or collisions with animals or previous vehicle/person damage in animal collisions. The alternative responses of acceleration (*n* = 2) and inaction (*n* = 9) were too infrequently reported to analyse. In avoiding an animal, 45% of respondents (including the majority of visitors and young people) would discriminate according to the size of the animal. When deciding whether to stop and check on the welfare of an animal victim, most people (67%) claim they would stop if they thought it would have a chance for survival, 46% would stop to check for pouch young (of a marsupial) and 45% (including the majority of respondents 60 years or older) would stop to check the animal if it was still visible from the road. Around 40% of respondents (including the majority of young people) said that whether they checked on the welfare of an animal or not depended on what type of animal it was (e.g., mammal, bird, reptile). A total of 83% of respondents claimed to drive slower where there are animals around (e.g., in wildlife zones). Over three times as many residents as visitors have wildlife deterrents fitted to their vehicles and more than twice as many residents as visitors carry provisions with them to help injured wildlife.

### 3.3. Driver Attitudes to Mitigating WVC

**Figure 3:**
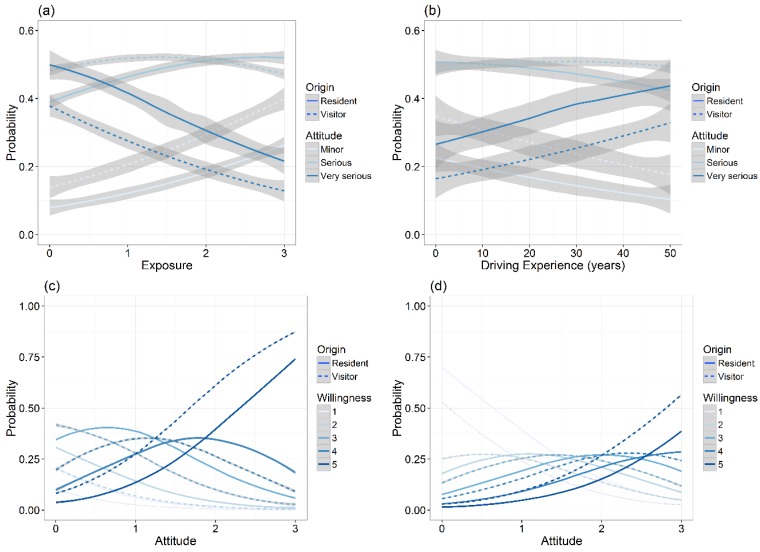
Modelled relationships of the predicted probability of questionnaire respondents considering road-kill to be a minor, serious or very serious issue in relation to increasing levels of (**a**) exposure or (**b**) driving experience, holding other variables at mean values. Similarly, willingness (5 = willing, 1 = unwilling) to adopt additional collision mitigations strategies of (**c**) driving slower or (**d**) reducing the amount of time spent driving at dusk and dawn with improving driver attitude was modelled from predicted relationships, holding other variables at mean values. Residential status (origin) is represented for residents and visitors as either ‘solid’ or ‘dashed’ lines respectively.

On average, respondents considered road-kill to be a serious or very serious issue (49% respondents). Improved attitude towards road-kill was higher for residents than visitors (significant at the 0.1 level) but significantly declined as exposure (experience of collisions and vehicle damage) increased (Table 1). While the likelihood that respondents considered WVC to be a serious issue did not change much with exposure, hovering at between 40% and 50% of respondents at both low and high levels of exposure, the likelihood that respondents considered WVC to be very serious significantly declined from around 40%–50% of respondents to 15%–25% of respondents as exposure increased (Figure 3). In contrast, driver attitude was unrelated to years of driving experience (Table 1) but there was a trend for those to view WVC as very serious to increase with experience. Respondents were most willing (average response: 4.5) to drive slower where there are animals around (e.g., in wildlife zones) and least willing (average response: 3.3) to drive less at dawn and dusk (approximately 6 am and 6 pm respectively during the study period) (Appendix B). On average, visitors were more willing to undertake the stated mitigation measures than were residents (with the exception of using a deterrent) and willingness also tended to increase with age. People that had never hit an animal were most willing to drive slower, while people that had hit more than ten animals were most willing to use a wildlife deterrent. Model testing showed that willingness to drive slower was significantly influenced by better driver attitude and by visitors, while exposure and experience were negatively correlated but not significant (Table 1). Likewise, willingness to avoid driving at dawn and dusk was shaped by better driver attitude and by visitors rather than exposure or experience.

### 3.4. Driver Speeds and WVC Risk

**Figure 4:**
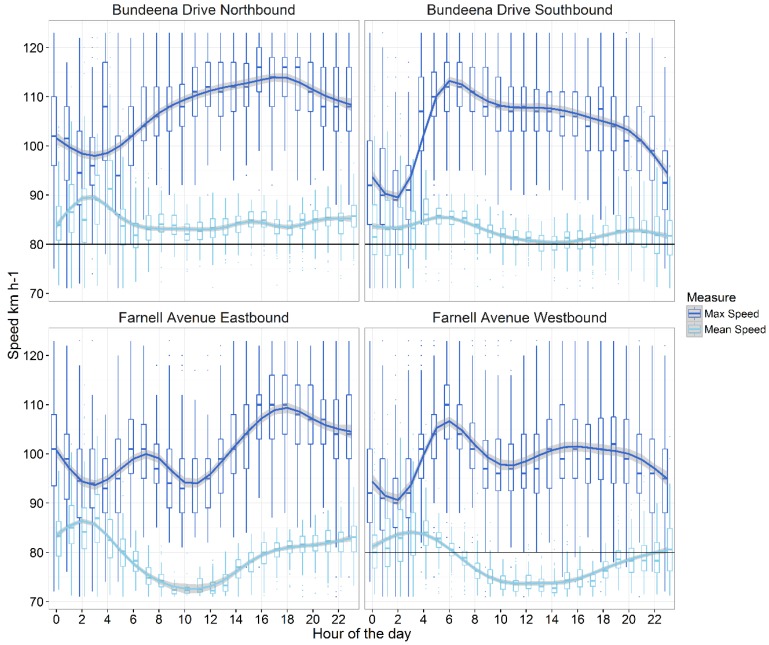
Maximum vehicle speed (mean hourly speed km·h^−1^, black line) and mean vehicle speed (mean hourly speed km·h^−1^, grey line) along Bundeena Drive near Bundeena Township and along Farnell Avenue near the entry to the Royal National Park between April and August 2003. Hourly boxplots and a smoothed loess conditional trend and standard error (shaded area) are represented for both, while the posted speed limit is depicted with a hashed line (80 km·h^−1^ except for Farnell Avenue Eastbound which was 60 km·h^−1^). Most upper and some lower outliers have been excluded to aid clarity.

We calculated average vehicle speeds consistently over the posted speed limit (Figure 4). Only vehicles exiting the national park during the middle of the day had consistent average speeds below the posted speed limit. In contrast, vehicles entering the national park in the early morning and late evening recorded average speeds up to 25 km·h^−1^ above the speed limit. Overall, vehicle speeds tended to peak in the early morning and rise in the evening when animals are most likely to be on the road. Hourly maximum speeds were consistently well above legal speed limits, with average maximums ranging between 95 and 110 km·h^−1^ on Farnell Avenue and between 90 and 115 km·h^−1^ on Bundeena Drive. These averages included regular transgressions of excessive velocity above 250 km·h^−1^, almost always at night.

## 4. Discussion

Most respondents to the questionnaire considered collisions with wildlife on roads to be a serious or very serious issue. Residents were generally more informed and had better attitudes towards WVC, a fact driven by their increased exposure to WVC. However, increasing exposure to WVC, and hence risks to drivers and their vehicles, resulted in a decline in the likelihood that respondents saw WVC as a very serious issue (Figure 3). This surprising trend is despite the fact that over half of the respondents had hit at least one animal during their time driving and almost as many had sustained some damage to their vehicle as a result, with increasing likelihood that damage would be substantial. This is also despite the fact that the strongest response from the questionnaire was that of the intention to drive slower where there are animals around, such as in posted wildlife zones. Respondents understood the message of wildlife warning signs about heightened risk of WVC and likewise understood the appropriate response of lowering risk by driving slower.

Responses about currently employed mitigation strategies and willingness to adopt additional mitigation strategies provided further insight into this finding. Braking was the most sensible avoidance option provided in the questionnaire and close to 80% of respondents stated that they reacted in this way. While this is perhaps a natural and logical response, it suggests a high degree of awareness among respondents. On the other hand, almost half of the respondents claimed to swerve in an attempt to avoid an animal. This is considered to be a dangerous option in Australia as the consequences of hitting an animal may be less severe than swerving to miss an animal and hitting something else instead, such as a tree on the verge or an oncoming vehicle. This suggests that many Australian drivers may still be unaware of the dangers of swerving, an issue of broader significance to road accidents than WVC, and further driver education may be required to address this issue. Other strategies, such as carrying provisions and phone numbers of wildlife carers, were less frequent and may reflect inhibition of taking action to assist wildlife even though attitudes towards WVC were generally good.

Willingness to modify current behaviours was similarly telling. Of the mitigation options provided in our questionnaire, respondents were generally most hesitant about reducing their amount of time driving at dusk and dawn. Most likely, this is due to the personal reduction of convenience this would cause, especially for local residents who may deem public transport insufficient (both Bundeena and Maianbar are accessible via a combination of train, bus and ferry), but also increasing familiarity with conditions. Visitors had significantly higher willingness to avoid dusk and dawn, although their motivations for driving at these times may be considerably less as many residents are employed outside the RNP. Only when respondents thought WVC was a very serious issue did willingness to avoid driving at dusk and dawn peak (Figure 4).

Drivers commit fewer violations and drive slower with increasing age [23,29], which implies that people tend to drive more sensibly as they get older. Thus if older drivers are more risk-averse, it follows that they would be more willing to take preventative measures against animal collisions. However, we found that respondents with good attitudes and less familiarity (*i.e.*, were visitors) were the most willing to adopt additional mitigation strategies. Although older people may have experienced more WVC incidents over their long driving careers, and the associated trauma (physical, emotional and/or financial), residents local knowledge that the risks of human injury and substantial damage from WVC is low may be resulting in complacency, and therefore reduced willingness to act.

This study examined not only what drivers say they do in relation to mitigating WVC but also provided an example how they act when confronted with the opportunity to reduce risk. Although we were unable to track the individual driving behaviours of respondents, we were able to identify general trends in driving behaviour. In the questionnaire, over 80% of respondents indicated that they minimised the amount of time they drove at night and that they drove slower in areas where wildlife were likely to be present. To verify whether these stated actions were put into practice we used traffic counters to measure the volume and speeds of vehicles at known areas of WVC (see [26]). Clearly, average and maximum speeds peaked during times it could be expected residents were going to and returning from employment outside the RNP. The contradictory results suggest many drivers are not risk-averse and other time-related priorities take precedence.

A similar contradiction between what drivers say and do has been reported for intentionality of collisions and avoidance of small fauna on roads [30]. While this may be of low consequence for WVC involving small animals, this is of concern when fauna increase in body weight. The most common species involved in collisions in the RNP likely to cause vehicle damage are swamp wallabies [26], with adult body weights up to 20 kg [31]. However, there are increasing numbers of Rusa deer [27] which can be up to 10 times the body weight of wallabies and pose a far greater risk to drivers [17]. Complacency towards WVC through past exposure and competing priorities may lead to drivers increasing their propensity for serious collisions [32].

In general, mitigation measures against WVC have been directed at wildlife, the engineering of roads especially verges, and various forms of warning about risk from general signage to contemporary warnings of greater immediacy [6,15]. Ultimately two parties come into play in a WVC. A driver, usually travelling at high speed, with variable attentiveness and reaction time and an animal, like a kangaroo, with unpredictable behaviour when confronted with the complex configuration of light, sound and movement from a vehicle. In general there is little that might increase the predictability of animal behaviour in advance of a vehicle [18]. Driver behaviour, however, can be controlled through legislative actions, education and engineering applications like traffic calming. However, in this study we found a high awareness amongst drivers of WVC and appropriate actions to avoid it but a low adherence to such actions.

## 5. Conclusions

While there is a wealth of research examining the causes of road accidents, we have yet to fully understand how drivers trade-off collision risk with wildlife against the knowledge that risk of serious damage or injury to drivers may be low. In Australia, where members of the family Macropodidae (e.g., kangaroos and wallabies) represent most large animals killed, collisions may number in the millions annually [33], yet human loss of life is rare [34]. In contrast, in countries where collisions with abundant megaherbivores are a serious safety concern for drivers, rather than primarily an environmental and wildlife welfare concern, willingness to modify behaviour may be being driven positively by exposure, unlike the negative relationships identified in this study.

## Figures and Tables

**Table 1:** Ordinal logistic regressions of driver attitude towards road-kill in relation to driver exposure, driving experience and residency status (expressed as visitor), and willingness to adopt additional collision mitigation strategies of driving slower or avoiding dawn and dusk driving.

Model	Variable	Value	SE	*t* Value	*p* Value	Odds Ratio
Attitude	Exposure	−0.479	0.224	−2.141	0.032 *	0.619
	Visitor	−0.631	0.341	−1.851	0.064	0.532
	Experience	0.019	0.012	1.523	0.127	1.019
Drive slower	Attitude	1.433	0.283	5.069	>0.001 *	4.191
	Exposure	−0.419	0.239	−1.754	0.079	−0.268
	Visitor	0.883	0.389	2.268	0.023 *	0.711
	Experience	−0.007	0.013	−0.491	0.623	0.001
Avoid dawn/dusk	Attitude	1.256	0.248	5.059	>0.001 *	3.512
	Exposure	−0.268	0.223	−1.203	0.229	0.765
	Visitor	0.711	0.333	2.135	0.033 *	2.036
	Experience	0.001	0.012	0.033	0.973	1.001

* Significant values at *p* < 0.05.

## Abbreviations

The following abbreviations are used in this manuscript:

WVC	Wildlife-vehicle collision
DOAJ	Directory of open access journals

## Appendix A

Driver Behaviour Questionnaire distributed to participants in the Royal National Park, Sydney, Australia. Bulleted points indicate available categories provided for each question. Wording has been modified for the purposes of publication. Space was provided for alternative answers where necessary.

1. Are you a visitor or local resident?
2. What is your age?
  - ≤20 years, 20–29 years, 30–39 years, 40–49 years, 50–59 years, ≥60 years.
3. What kind of vehicle(s) do you normally drive?
  - 4-wheel drive *or* people mover, family sedan, utility *or* small van, motorcycle, sports car, articulated truck, light truck, bus, heavy rigid truck, other.
4. How many years have you been driving for?
  - <1 year, 1–3 years, 4–9 years, 10–19 years, 20–29 years, 30–39 years, 40–49 years, ≥50 years.
5. In the time you have been driving, around how many animals have you hit?
  - None, none hit but some near misses, 1–10, More than 10.
6. Of those animals you have hit (or nearly hit), what proportion were native Australian animals?
7. In most cases, if you saw the animal before hitting it, what did you do?
  - Swerved, braked, accelerated, took no action.
8. If you took avoidance actions what did this depend on?
  - The size of the animal, the type of animal (reptile, bird, mammal), the status of the animal (native or introduced).
9. What circumstances would cause you to check on the welfare of the animal (regardless of who hit it)?
  - None, the animal was still visible from the road, I had free time, I thought it had a chance for survival, I thought it might have pouch young.
10. If you did check on the welfare of the animal, what did this depend on?
  - The size of the animal, the type of animal (reptile, bird, mammal, *etc.*), the status of the animal (native or introduced).
11. Do you currently do anything to minimise your chances of hitting or killing an animal?
  - Travel slower where there are animals around (e.g., in signposted wildlife zones), attach wildlife deterrents (such as whistles) to your car, carry provisions in your vehicle to help injured wildlife (e.g., gloves, blanket), keep the phone numbers of wildlife rescue organisations (e.g., WIRES) with you, other.
12. What damage has your vehicle ever sustained in a collision (or near collision) with an animal?
  - Minor damage, substantial damage (e.g., insurance claim), vehicle unrepairable, persons injured.
13. How do you feel about the issue of road-kill?
  - It is a very serious problem, it is a serious problem, it is a minor problem, no problem at all, indifferent.
14. On a scale of 1–5, how willing would you be to take the following actions to minimise road-kill (1 = totally against it, 2 = unwilling, 3 = hesitant, 4 = willing, 5 = happy to)?
  - Drive slower, reduce speed in wildlife crossing zones, reduce amount of time driving at dusk and dawn, reduce speed at dusk and dawn, use a wildlife deterrent for your car that is cheap and proven to be effective.

## Appendix B

Summary of driver responses to questionnaire. Values are proportions of each respondent type who gave the response, with bold indicating that more than half of those respondents answered in that way. The last two response categories note the average rating of each response by each respondent type, with bold highlighting the highest average ratings of each response within each respondent category. (Survey question numbers being referred to are in square brackets while the number of respondents in each category are in parentheses).

**Table B1:** Summary of driver responses to questionnaire.

Question	Category	Origin		Age			Animals Hit				
		Resident (105)	Visitor (51)	<30 (24)	30–59 (111)	>59 (18)	None (42)	Near Miss * (34)	1–10 (76)	>10 (3)	Total (156)
**Vehicle type [3]**	4WD	0.24	0.25	0.13	0.23	0.44	0.14	0.38	0.24	0.33	0.24
	Ute/Van	0.06	0.10	0.08	0.06	0.06	0.02	0.03	0.11	0.33	0.07
	Art. Truck	0.00	0.00	0.00	0.00	0.00	0.00	0.00	0.00	0.00	0.00
	HR Truck	0.01	0.00	0.00	0.01	0.00	0.00	0.03	0.00	0.00	0.01
	Sedan	**0.53**	**0.57**	**0.58**	**0.58**	0.39	**0.62**	**0.56**	**0.51**	0.00	**0.54**
	Motorbike	0.03	0.02	0.04	0.02	0.06	0.00	0.03	0.03	0.33	0.03
	Light Truck	0.02	0.00	0.00	0.02	0.00	0.05	0.00	0.00	0.00	0.01
	Compact	0.08	0.08	0.17	0.06	0.06	0.10	0.00	0.11	0.00	0.08
	Sports	0.03	0.02	0.08	0.02	0.00	0.05	0.06	0.00	0.00	0.03
	Bus	0.00	0.00	0.00	0.00	0.00	0.00	0.00	0.00	0.00	0.00
	Other	0.08	0.04	0.08	0.05	0.06	0.02	0.00	0.11	0.33	0.06
**Damage sustained in animal collision [12]**	Minor	0.33	0.27	0.46	0.30	0.28	0.12	0.18	0.47	**0.67**	0.31
	Substantial	0.19	0.06	0.08	0.15	0.17	0.00	0.09	0.24	**0.67**	0.15
	Total (write-off)	0.02	0.02	0.00	0.02	0.06	0.00	0.03	0.03	0.00	0.02
	Persons injured	0.04	0.02	0.04	0.04	0.00	0.00	0.06	0.04	0.00	0.03
**Reaction to animal on road [7]**	Swerved	**0.52**	0.43	**0.54**	0.49	**0.50**	**0.52**	0.41	**0.50**	**1.00**	0.49
	Braked	**0.79**	**0.76**	**0.58**	**0.81**	**0.83**	**0.81**	**0.91**	**0.70**	**1.00**	**0.78**
	Accelerated	0.01	0.02	0.00	0.02	0.00	0.00	0.00	0.03	0.00	0.01
	No action	0.06	0.04	0.04	0.05	0.06	0.00	0.03	0.09	0.00	0.05
**Animal avoidance criteria [8]**	Size	0.42	**0.51**	**0.58**	0.42	0.44	0.48	**0.56**	0.38	**0.67**	0.45
	Type	0.27	0.25	0.25	0.26	0.28	0.21	0.29	0.28	0.33	0.26
	Status	0.25	0.22	0.21	0.23	0.28	0.19	0.26	0.24	0.33	0.24
**Situation criteria for stopping to check on animal [9]**	Would not check	0.02	0.04	0.04	0.02	0.06	0.05	0.00	0.03	0.00	0.03
	Visible from road	0.43	0.49	0.46	0.43	**0.56**	0.40	0.44	0.46	**1.00**	0.45
	Spare time	0.10	0.08	0.17	0.09	0.00	0.02	0.12	0.12	0.00	0.09
	Chance for survival	**0.67**	**0.69**	**0.67**	**0.70**	0.44	**0.64**	**0.65**	**0.68**	**1.00**	**0.67**
	May have pouch young	0.48	0.43	0.46	0.46	0.44	0.40	**0.53**	0.45	**1.00**	0.46
**Animal criteria for checking welfare [10]**	Size	0.22	0.25	0.25	0.23	0.28	0.24	0.26	0.22	0.00	0.23
	Type	0.39	0.43	**0.54**	0.41	0.22	0.31	0.41	0.45	**0.67**	0.40
	Status	0.31	0.31	0.42	0.26	0.50	0.29	0.38	0.29	0.33	0.31
**Current mitigation measures [11]**	Drive slower in wildlife zones	**0.79**	**0.92**	**0.63**	**0.88**	**0.78**	**0.88**	**0.85**	**0.80**	**0.67**	**0.83**
	Use deterrent	0.10	0.04	0.04	0.05	0.28	0.02	0.12	0.09	0.00	0.08
	Carry provisions to help animal	0.17	0.08	0.17	0.14	0.11	0.14	0.15	0.14	0.00	0.14
	Carry wildlife rescue phone no.	0.27	0.25	0.25	0.27	0.22	0.31	0.24	0.22	**0.67**	0.26
	Other	0.21	0.06	0.29	0.14	0.06	0.17	0.12	0.18	0.00	0.16
**Willingness to undertake mitigation ** [14]**	Drive slower	4.2	**4.5**	4.2	4.3	**4.5**	**4.6**	4.4	4.1	3.5	4.3
	Drive slower in wildlife zones	4.5	**4.7**	4.4	4.6	**4.7**	**4.8**	4.6	4.4	4.0	4.5
	Drive less at dawn & dusk	3.2	**3.6**	3.3	3.4	**3.8**	3.4	**3.6**	3.2	2.0	3.3
	Drive slower at dawn & dusk	4.1	**4.5**	4.1	4.3	**4.3**	**4.4**	4.3	4.1	3.3	4.2
	Use deterrent	**4.4**	4.3	4.5	4.5	**4.6**	4.3	4.6	4.3	**4.7**	4.4

* No animals hit, but some near misses; ** On a scale of 1–5, where 5 = happy to and 1 = totally unwilling to.
